# Volunteer Engagement in a Stroke Self-Management Program: Qualitative Analysis of a Hybrid Team of Healthcare Providers and Trained Volunteers

**DOI:** 10.3390/ijerph19159341

**Published:** 2022-07-30

**Authors:** Suzanne Hoi Shan Lo, Janita Pak Chun Chau, Ravneet Saran

**Affiliations:** The Nethersole School of Nursing, Faculty of Medicine, The Chinese University of Hong Kong, Hong Kong SAR, China; janitachau@cuhk.edu.hk (J.P.C.C.); ravneet.sar@gmail.com (R.S.)

**Keywords:** stroke, volunteers, self-management, rehabilitation, self-efficacy

## Abstract

Stroke recovery is a complex, multidimensional and heterogeneous process. Volunteer engagement improves the delivery of interventions in stroke rehabilitation programs but is under-utilized due to poor role clarity and other program-related concerns. We evaluated healthcare providers’ and volunteers’ perceptions of volunteer engagement in an 8-week self-management program that provided self-management support for community-dwelling stroke survivors. Using a qualitative design, we conducted individual, semi-structured interviews with a purposive sample of 5 trained healthcare providers and 18 volunteers. The participants shared their experiences of supporting survivors, perceptions of volunteer engagement, and areas of improvement to optimize volunteer support. Three main themes and six subthemes emerged: bilateral exchange between healthcare providers/volunteers and survivors; adoption of individualized approaches; and suggestions for optimizing volunteer contributions. Volunteer engagement can be optimized by developing well-designed programs with sufficient role clarity, strengthened collaborations with healthcare providers and adequate training. Our findings highlighted the contributions of trained volunteers in supporting stroke survivors’ self-management. Future research should evaluate the use of peer and healthcare professional volunteers in such programs and build community capacity to support stroke survivors’ recovery.

## 1. Introduction

Stroke is a leading cause of chronic adult disability globally. As of 2019, new stroke incidence has grown by 13.7 million [1]. After stroke, survivors are commonly left with a variety of inter-related physical, cognitive and psychosocial sequelae that adversely impact their health-related quality of life and social participation [2]. Such post-stroke consequences continue to afflict community-dwelling stroke survivors long after they are discharged from the hospital, with many reporting mobility issues and extreme difficulties in performing household and self-care activities [3,4]. Previous research has also widely highlighted the detrimental effects of other modifiable risk factors such as persisting psychological distress, fatigue and social isolation in community-dwelling stroke survivors, which may last for more than 12 months after the stroke event [5]. Effective stroke rehabilitation programs that target community-dwelling stroke survivors are crucial to help them overcome these challenges and facilitate recovery and reintegration into the community.

Stroke recovery is a complex, multidimensional and heterogeneous process. Guidelines from the American Heart Association/American Stroke Association stress the importance of coordinated efforts by a large, multidisciplinary team of healthcare professionals in tandem with stroke survivors’ personal goals, family members and caregivers [6]. Recently, the use of stroke self-management programs to improve survivors’ activities of daily living (ADLs) and community reintegration and reduce their dependence has received much support [7,8]. Self-management has been defined as an individual’s ability to manage the symptoms, treatment, physical and psychosocial consequences and lifestyle changes inherent in living with a chronic disease [9]. Stroke rehabilitation encourages survivors to be more active in setting goals and in their recovery journeys. Other core elements of self-management are problem solving, decision making, action taking and using available resources [9]. Increasing evidence has shown the association of self-management programs with significant improvements in self-efficacy, quality of life, social participation and the management of stroke symptoms in stroke survivors [10,11]. Given the integral role of self-management interventions, it would be valuable to integrate them into tailored stroke programs to promote the survivors’ recovery.

Volunteering is a promising strategy that has had positive effects on the management of stroke and other chronic conditions. It is an approach wherein volunteers support clients and enhance the clients’ well-being via health education and promotion [12,13]. Volunteers in stroke programs contribute to a variety of areas by directly interacting with stroke patients and offering administrative support [14]. It has also been reported that volunteer engagement in stroke programs strengthens intervention delivery by ensuring that important health messages are effectively disseminated to clients. Through the use of lay language, volunteers, particularly those from the general public, enhance the immediate applicability of delivered health information for clients and minimize barriers in understanding that may arise from professional distance between healthcare professionals and clients [15,16]. Moreover, previous evidence has highlighted the benefits of participation for the volunteers themselves, who have reported enhanced learning, understanding and empathy after interacting with clients [15]. Volunteers have increasingly played vital roles in health promotion and the rehabilitation support of people, including stroke survivors. Despite the cost-effectiveness and overall effectiveness of volunteer engagement, volunteers remain under-utilized in stroke programs due to a lack of clarity in delineating their distinct responsibilities and concerns of poor attendance [14]. There has been little research on the volunteers’ experiences of assisting stroke survivors in self-management. More research on well-planned stroke interventions involving volunteers is important to enrich existing stroke rehabilitation programs.

The authors (SHSL and JPCC) developed an 8-week enhanced stroke self-management program (Coaching Ongoing Momentum Building On stroKe rEcovery journeY [COMBO-KEY]) that aimed to provide self-management support for community-dwelling stroke survivors through home visits and phone coaching [17]. It featured a hybrid team of trained coaches, including healthcare providers who served as leaders, and volunteers who served as coaches to support program implementation. The program consisted of eight weekly sessions, with four home visits by the healthcare leader and five follow-up phone calls by the volunteer. The healthcare leader set a short-term goal of recovery and an action plan with the participating stroke survivor. In addition to providing health information commonly adopted in traditional rehabilitation programs, the stroke survivor was guided in performing self-management behaviors and exercising self-management skills such as goal-setting and problem-solving skills. Theory-driven strategies were adopted to increase the survivors’ self-efficacy and outcome expectation of performing self-management behaviors. Each survivor was provided with access to a website with tailored information about stroke self-management and videos of peer survivors’ sharing of survival experiences [17].

In this study, we evaluated the healthcare providers’ and volunteers’ perceptions of volunteer engagement in COMBO-KEY. We sought to understand their perceptions of the gains from and challenges to collaborating to support survivors through the program and areas in the program that could be modified to optimize volunteer support for the survivors. Findings from this study can help to understand volunteers’ contributions to and needs for supporting survivors’ self-management and hence provide insights into the effective design and delivery of volunteer-assisted self-management programs to enhance survivors’ recovery outcomes.

## 2. Materials and Methods

### 2.1. Study Design and Participants

A qualitative research design comprised of individual semi-structured interviews of healthcare providers and volunteers participating in COMBO-KEY was adopted. The study met the Consolidated Criteria for Reporting Qualitative Research [18]. A purposive sample of 23 interviewees who met the following inclusion criteria was recruited from various settings in Hong Kong such as universities, hospitals and non-governmental or community health organizations: (1) aged 18 or above, (2) able to read and communicate in Cantonese, (3) studying for or obtained a bachelor’s degree in health or social sciences, and (4) delivered phone coaching and/or home visits to stroke survivors participating in COMBO-KEY.

The interviewees had a mean age of 28.74 years (standard deviation [SD] = 9.16, range = 21–50 years). Most of them were under 30 years old (*n* = 18, 78%) and female (*n* = 18, 78%). Of the healthcare providers, three (60%) were registered nurses, and two (40%) were social workers. They had a mean work experience of 13 years (SD = 7.81). Of the 18 volunteers (mean age = 26.94 years; SD = 8.73), the majority (*n* = 10, 55.6%) were pursuing a health-related bachelor’s degree. The most common major was nursing (*n* = 7, 70%), followed by gerontology (*n* = 2, 20%) and psychology (*n* = 1, 10%). The remaining eight volunteers (44.4%) had full-time jobs; among them, three (26%) were registered nurses, one was a social worker (13%), two were research assistants (9%) and two were clerks (9%). Most of the volunteers (71.4%) supported 1–5 clients. The clients (*n* = 67, mean age 63.4 years, SD = 14.1) had a mean duration of stroke onset of 4.1 years (SD = 3.9). Nearly one-fourth of them walked unaided and the others were able to walk with a stick or a walking frame. All of them were able to provide informed consent to participate in the program, communicable in Cantonese with the healthcare providers and volunteers, and did not have a diagnosed mental condition. Table 1 summarizes the interviewees’ demographic characteristics.

### 2.2. Data Collection

A semi-structured interview guide (Table 2) was developed based on the results of literature reviews and the project team members’ experience. A trained research assistant experienced in conducting qualitative interviews carried out individual, semi-structured interviews with the eligible participants over the phone. All of the interviews were conducted at times convenient for the participants, between February 2021 and May 2022. The participants shared their experiences of supporting survivors in COMBO-KEY, perceptions of volunteer engagement in the program, gains from and challenges to collaborating for supporting survivors in the program and areas of improvement to optimise volunteer support to the survivors. All of the interviews lasted 30–45 min, were conducted in Cantonese and were audio-recorded and duplicated to avoid accidental data loss. Volunteers’ demographic and work characteristics, such as age, gender, education level and occupation, were recorded.

The principal investigator (PI) was a female registered nurse and Ph.D. holder with extensive experience in stroke rehabilitation, qualitative studies and semi-structured interviews. The research assistant (RA) was a male with a bachelor’s degree in gerontology. The two interviewers had no prior relationships with the participants. The PI provided the RA with a 1-day training module covering telephone-based interviewing skills, research integrity and communication with survivors. Role-plays of interviewing a potential participant were conducted with the RA. The RA was supervised by the PI when conducting the first two interviews and received feedback from the PI to ensure satisfactory interviews. The RA reported on his progress to the PI on a regular basis and received continuous feedback on his performance.

### 2.3. Data Analysis

All of the interview data were transcribed verbatim from audio recordings. The interview transcripts were anonymized by substituting participant numbers and reviewed independently by a RA (RS) for accuracy. As the pilot interview was rich in information, the data were included in the analysis. The first (LSHS) and second (JPCC) authors analyzed the transcripts independently following the six phases of thematic analysis [19]. The two authors first read the transcripts several times to familiarize themselves with the data. Initial codes were created by inductively coding the transcripts line by line, in accordance with the aim of the study. After re-verifying the initial codes against the data, a preliminary coding scheme was developed. Similar codes were further grouped into subthemes and broader themes. The themes and subthemes were carefully reviewed to ensure clarity and coherence with one another and the study aims. Finally, the theme and subtheme names were deliberately considered and refined to ensure that they were clear, concise and best represented the concepts being described. Disagreements in interpretation were resolved through discussion among the three authors. Illustrative quotes were chosen from some of the participants to outline the respective themes and subthemes; these quotes were translated from Traditional Chinese to English by the first author (SHSL), who was fluent in both languages. The participants’ information was provided as Reference Number_Role in the program (provider or volunteer).

### 2.4. Ethics and Consent 

This study was approved by the Joint Chinese University of Hong Kong-New Territories East Cluster Clinical Research Ethics Committee (Reference no.: 2018.009). All of the participants received a full explanation of the study prior to data collection and were informed of their rights to refuse participation or withdraw from the study at any time. Written informed consent was obtained from all of the participants. All of the data collected were anonymized, used for research purposes only and kept strictly confidential in a locked cabinet or via encryption, with access granted only to research team members. Local laws, the Declaration of Helsinki, institutional policies and the International Conference on Harmonization-Good Clinical Practice were strictly adhered to throughout the study.

### 2.5. Research Integrity

Strategies such as member check (credibility), thick description (transferability), audit trail (dependability and confirmability) and journaling (reflexivity) were adopted to maintain rigor and trustworthiness [20]. The interviewees were provided with the transcripts of their interviews and asked to give feedback to ensure correct interpretations of their perceptions. A detailed description of the data collection process and context was provided. By documenting all the coding decisions agreed upon by the research team, we maintained a transparent audit trail. The team members who analyzed the data regularly referred to the audio files to ensure accuracy. During data collection, all interviewers maintained a reflexive journal to record their feedback and any issues they thought might affect data analysis. The reflexive journals were discussed in the research team’s regular meetings for data interpretation

## 3. Results

Three themes and six subthemes emerged from the interview data (Table 3).

**Theme 1: Bilateral exchange between survivors and healthcare providers/volunteers**

All healthcare providers and volunteers consistently described their experiences supporting stroke survivors in COMBO-KEY as rewarding. The volunteers recalled that they chose to participate in the program primarily because they ‘hoped to help others and help themselves’. They wanted to use their knowledge and skills to help others and to meet other people sharing the same goals; these aims were largely achieved after completing the program. Most of the volunteers regarded their volunteering experiences as resembling a mutual learning experience between themselves and the survivors. They exchanged and learned pragmatic tips for self-managing a stroke and life management wisdom for embracing life challenges and maintaining positive momentum. Bilateral exchanges also existed between the healthcare providers and volunteers in terms of professional knowledge and skills and positive attitudes towards caring for survivors.

Subtheme 1.1: Exchanging information about care after stroke

The volunteers usually reinforced stroke care information such as lifestyle modifications and principles of self-management during the program. As they were providing the information, over half of the volunteers recalled that some of the survivors either asked them for information about stroke, for which the volunteers needed to review current evidence, or asked for community services, about which the volunteers did not have knowledge, needed to search and/or consult healthcare providers. The volunteers then responded to the survivors; the volunteers regarded this process as allowing them to consolidate their knowledge of stroke and improve their understanding of the survivors’ health needs.

*I was asked whether it was appropriate to take a Chinese herbal medicine when there were signs of recurrent stroke…whether that vegetable can lower blood pressure…honestly…I needed to search for info…ask my leader [healthcare provider].*

(P10_Volunteer)

*One survivor wanted to join exercise classes…convenient…not expensive…within walking distance…there was a lot of info…I screened…prioritized…took me a few hours…I have a clear idea now.*

(P15_Volunteer)

Volunteers who were students of health-related disciplines added that they could practise the health-related skills they learned from classrooms in a hands-on manner. One volunteer shared the following:

*When in practice, I need to express my content [health info] in a way that they [survivors] understand, accept and remember…I have to double check the info…make sure it is correct…I learned to apply the knowledge to help a person’s recovery.*

(P6_Volunteer)

The volunteers unanimously agreed that the survivors sometimes shared how they managed to tailor their living environments or daily activities to facilitate self-care or physical activities. The volunteers were impressed by the tips, which were useful, innovative, and personalized for self-managing a stroke and not readily available from books or journals.

*A survivor told me…he hangs a ball from the ceiling, so he can kick the ball and do lower limb exercises…it’s a smart idea…the ball comes back automatically.*

(P18_Volunteer)

A bilateral exchange of health information was also perceived between the healthcare providers and volunteers. Three volunteers expressed concerns about their competency in supporting survivors’ self-management at the beginning of the program. After knowing that a healthcare professional would lead them in providing support and guidance and advice, they said that they were relieved as they could know whether they were ‘on the right track’. A volunteer who was a nursing student shared the following:

*I learned a lot from her [leader]…communication skills, practical strategies for survivors’ self-care, updated knowledge…even how to interact with survivors, tips and precautions during home visits and phone calls.*

(P6_Volunteer)

Another volunteer stressed the importance of having a healthcare professional give them accurate information to ensure that the volunteer was treating the survivors well.

*Just having a good heart is not enough…I learned something (during training)…but not enough…these people [survivors] may have some unique needs…also may change over time.*

(P11_Volunteer)

The healthcare providers reciprocally appreciated the contributions of the volunteers. One shared her experience of learning from a volunteer:

*Self-management can be broad and individual…they [volunteers] are good at web surfing…studying in health…my volunteer searched for some information about choice of food…I learned something new.*

(P1_Provider)

Subtheme 1.2: Being inspired by life management wisdom and professional attitudes

The volunteers recalled that most of the survivors liked to chat with them about the changes in their lives after stroke as well as feelings and hardships. They agreed that most of the survivors valued having a person listen to their thought and feelings.

*She [survivor] thanked me for listening to her, it had been long since someone listened to her for a long time. She needed a way of expressing herself and felt more relieved.*

(P13_Volunteer)

The volunteers enjoyed chatting with the survivors during the programme. The volunteers mentioned that they provided positive reinforcement and encouragement to survivors to participate in the self-management and recovery process. Reciprocally, they learned from the survivors about perseverance in addressing post-stroke challenges.

*One survivor, aged around 60, told me no matter what…painful or not, he will persist in working hard at rehab exercises, this is what he has to do…for his own good…I should be that strong in working out my professional challenges.*

(P17_Volunteer)

*Sometimes I felt embarrassed when I asked them to work hard, think positive…looking at myself…I was hesitant in facing my difficulties in my study.*

(P21_Volunteer)

The survivors sometimes shared with volunteers their past life history and achievements, or what they experienced in their younger lives. They shared their life management wisdom, which the younger volunteers found particularly helpful.

*She [survivor] told me how she worked from an apprentice to a supervisor…she learned English by self-study at night…how her colleagues tried to stop her from getting promotion…how she worked through these…just like a mini-mentoring session….*

(P22_Volunteer)

The volunteering experiences also allowed the healthcare providers and volunteers to feel supported and like a team working towards a common goal. Two healthcare providers expressed that they were at times positively influenced or motivated by the passion of the volunteers.

*They [volunteers] wanted to serve, contribute…eager to learn…I was once asked (by volunteers) about how to better respond to the survivors’ concerns…this simple question reminds me of our goal of volunteering…gave me momentum to continue.*

(P2_Provider)

The volunteers also appreciated the support given by the healthcare providers. Seven of them said that they were inspired by the healthcare providers’ positive attitudes towards supporting the survivors.

*I realised how a caring nurse should behave…She [leader] was not only good to people with stroke, but also taught me with patience, I feel the difference the care makes…deepened my interest in community nursing.*

(P10_Volunteer)

**Theme 2: Concerted efforts in guiding and motivating survivors**

The volunteers shared that, prior to this study, they chiefly participated in one-off services for groups of people that lasted for 2–3 h. They found the duration and frequency of COMBO-KEY to be relatively intense. In addition, despite over half of the volunteers pursuing a health-related discipline, they consistently found it challenging to apply personalised care strategies that aided survivors, helped them work towards their action plans and thereby attain their goals. However, the volunteers, regardless of background, acknowledged the importance of personalizing recovery goals and strategies to raise the confidence of survivors. They recognised the uniqueness of individuals with stroke and agreed that learning to effectively master self-management behaviors takes time. They unanimously expressed their passion for guiding and motivating the survivors to set and attain goals and increasing survivors’ confidence in self-management.

Subtheme 2.1: Keeping survivors on track with their goals

Most of the volunteers found their involvement in goal setting as one of the most helpful aspects of the regular interventions provided by the program. They shared some noticeable improvements in the survivors’ physical and social functioning over the course of the program. They were satisfied with their efforts.

*He [survivor] resisted talking to me at the beginning…I kept suggesting methods by which he could reach the goal...in the third call, he told me happily that he felt his fingers move better…he wanted to have another goal.*

(P8_Volunteer)

*I feel she [survivor] needs a direction…every time I called her, she said “I did the homework”…she then told me how she could walk better that week…I would say “great job”.*

(P10_Volunteer)

The majority of the volunteers said that they followed up with the survivors about their progress in achieving the established goals; information about the goal was conveyed to the volunteers by the healthcare providers, thus ensuring that the volunteers generally followed the care plans and faced no major difficulties in guiding the survivors to work towards the goals.

*My leader passed me the survivor’s plan and goal… explained to me what to focus on…I just needed to follow and get back to my leader if anything went wrong or if I had any queries. Most of the time, it worked smoothly.*

(P8_Volunteer)

All of the healthcare providers echoed the view that the volunteers were capable of working as part of a trusted team and guided survivors towards their goals and action plans.

*I think my volunteer did a great job…he paid attention to what I highlighted and guided the survivors in the same direction.*

(P1_Provider)

Three healthcare providers added that the volunteers without a health background required more supervision and guidance in carrying out plans for the survivors at the beginning of the program. For example, some volunteers commonly mixed up positive outcome expectations and goals and required multiple clarifications. Furthermore, one or two volunteers needed intermittent supervision and progress checks in subsequent sessions, depending on their abilities. Commonly reinforced bits of information were the principles of the SMARTER (Specific, Measurable, Achievable, Relevant, Timely, Evaluate, Revise) goal system, importance of the goals, how they related to the survivors’ recovery and questioning skills to determine the difficulties faced by survivors in attaining their goals. Despite this, the healthcare providers consistently acknowledged the contributions of the volunteers in supporting the survivors and building community capacity.

*I don’t mind spending more time in sharing knowledge with my volunteers…they are also willing to know more…if they know more, they can offer better care, which eventually benefits our survivors.*

(P2_Provider)

*We really need more people in the community to support the survivors…their needs are long-term…they should not be left behind after stroke…experience of helping can be accumulated…the volunteers are doing better and better.*

(P1_Provider)

When asked about the impressive or challenging aspects of guiding and motivating survivors, two healthcare providers said that the volunteers spent significant amounts of time discussing with the survivors the details of what they actually wanted to do and giving feedback. This then facilitated refinements of the short-term goal. One healthcare provider shared the following.

*My volunteer told me about the survivor’s route to get to the workplace before stroke…they had concerns about getting onto the bus…we modified the goal to train his lower limb strength and balancing ability...we reinforced his picture of the goal from going outdoors to resuming work to earning money…more motivation.*

(P1_Provider)

Subtheme 2.2: Personalizing care to raise confidence

Nearly all volunteers mentioned that the program was characterized by its emphasis on individualising strategies to increase survivors’ confidence in performing self-management behaviors. When asked about the strategies commonly used in this regard, the volunteers consistently mentioned providing positive reinforcement and acknowledging incremental efforts and/or achievements. All of the volunteers actively complimented survivors on their progress throughout the program, which encouraged and increased their confidence in working towards their goals.

*I feel like they need someone to acknowledge their hard work…someone should say their hard work is on the right track, I usually say they are doing well…they then feel more comfortable to keep going.*

(P10_Volunteer)

In contrast, a few volunteers doubted the extent to which their positive reinforcement could build survivors’ confidence.

*My survivor seems to not accept my general positive appraisals, like ‘well done’…I a bit feel embarrassed…but just don’t know what else I can say.*

(P19_Volunteer)

Some volunteers highlighted the usefulness of videos showing other survivors in similar situations; these videos helped survivors think more positively and develop a sense of belonging with other survivors, raising their confidence in overcoming their post-stroke challenges.

*Not sure if I am too young, I don’t seem persuasive enough to ask them to be confident in getting through this critical condition [stroke]…the peers’ survival experience videos provide strong words.*

(P9_Volunteer)

The volunteers also indicated that they attempted to adapt the interventions or care provided to each survivor’s specific concerns, emotions and interests. They put considerable effort into asking and understanding the survivors’ difficulties and hesitations, identifying personalized strategies to attain goals and building confidence in self-management. However, both the healthcare providers and volunteers agreed that it required further testing and was not always successful.

*I tried to encourage her [survivor], adjust the schedule of exercise…suggest ways of rearranging her household things to create space for exercise…but each session keeps requiring adjustments….*

(P9_Volunteer)

A healthcare provider echoed this observation:

*No single method applies to all people…just keep trying, accumulate experience, you will have more methods in your pocket for different survivors.*

(P1_Provider)

**Theme 3: Suggestions for optimizing the contributions of volunteers**

When asked about areas for further improvements to volunteering experiences, the healthcare providers and volunteers mainly focused on clarifying the others’ roles and responsibilities, strengthening collaborations and better equipping the volunteers to handle various facets of the survivors’ problems.

Subtheme 3.1: Clarifying the importance of roles and collaboration

The lack of steady availability of volunteers at different periods of time in the program was raised as a common barrier to optimizing volunteer support. The healthcare providers acknowledged the limitations of volunteering and the difficulty in maintaining a steady, core team that could provide support whenever needed. The volunteers also admitted that they had to place their jobs or study-related commitments at a higher priority than the volunteering services.

*They [volunteers] have a full-time job or study and sometimes are not available or too tired to take up volunteering for the stroke survivors when needed.*

(P5_Provider)

*I stopped volunteering for about 3 months during my examination period and re-joined after exams.*

(P10_Volunteer)

Some healthcare providers expressed concerns about the continuity of services and changes to the collaborations between themselves and volunteers due to the changing availability of volunteers.

*A volunteer needed to stop in the middle of the program…I needed to arrange and work with another volunteer…but rapport with survivors is important for this kind of personalized program.*

(P5_Provider)

One healthcare provider mentioned that this problem might arise from the volunteers’ lack of understanding of their roles and the importance of the collaboration between themselves and the providers.

*I felt like some volunteers thought that they were only doing follow-up phone calls and that the key role of support lies with the healthcare provider….*

(P1_Provider)

One volunteer echoed this observation:

*My main role is to encourage the survivor to work towards the goal…and report any abnormalities to my leader…if there is nothing abnormal, I seldom bother my leader.*

(P20_Volunteer)

It was suggested that the volunteer–provider collaboration could be improved if either the healthcare provider or volunteer, or both, consistently conducted both the home visits and phone coaching.

*It would be better if the same person or persons could work with one case…do both home visits and phone calls…maintain consistency and build a [closer] relationship.*

(P16_Volunteer)

*Sometimes they [volunteers] may be shy…actually we have not known each other for long…we need more common experiences…opportunities to discuss and share.*

(P1_Provider)

Another healthcare provider highlighted the importance of recruiting and training more people to serve as volunteers to care and support the survivors and improve the stability and continuity of support.

Subtheme 3.2: Fulfilling training needs in caring for survivors

Most of the volunteers mentioned that they did not feel sufficiently prepared to deal with diverse situations, such as verbally responding to the survivors’ feelings and questions about stroke care over phone calls or being sensitive enough to identify the survivors’ difficulties, concerns or other problems, given their unique health needs. The volunteers suggested that it would be beneficial to allocate more time to the training period to enhance their caring skills. This would allow them to play a more active role in promoting the survivors’ self-management capabilities and recovery. Common topics that were recommended for inclusion in the training module were dealing with emotional problems, stroke prevention strategies and rehabilitation interventions:

*During the phone calls, I worry about how their emotions may change…they may see me as a professional and I feel like I need to know more about stroke rehab to engage in conversations with them in a relevant and helpful manner.*

(P11_Volunteer)

The provision of self-management support sometimes requires information about the availability of community support services. About one-third of the volunteers, mostly those pursuing health-related degrees, mentioned that they provided information about stroke support groups, community-based organizations offering rehabilitative, social or vocational services, meals-on-wheels services, private physiotherapy services and leisure activities. They recommended maintaining an updated list of such community-based services and information on referrals to inform the survivors.

*I can search for the info…but a readily available set would be better…we should make sure it is correct…also, referrals are not easy to find.*

(P23_Volunteer)

Another common challenge lay in communicating effectively with survivors and understanding what they said during a phone call. The challenge was intensified when the stroke survivors had dysphasia or slurred speech. The volunteers expressed a desire for longer training periods to attain the skills necessary to appropriately initiate and manage conversations with stroke survivors and learn special communication techniques for survivors with speech problems. They also highlighted the need to reinforce volunteers’ speaking skills and abilities to guide volunteering sessions through, for example, the provision of guiding questions for different scenarios.

*We need more training on how to start talking about different topics with the stroke survivors…we need to develop skills to invite them to talk…more practice is needed to apply this knowledge…and how to tailor conversations to survivors with different personalities.*

(P23_Volunteer)

One volunteer suggested that working with peer volunteers in such stroke programs may help strengthen the volunteers’ first-hand understanding of stroke survivors and better support the survivors.

*The program should provide more opportunities for us to talk and work with other volunteers from stroke support groups…We can then have a more comprehensive and deeper understanding of the survivors.*

(P18_Volunteer)

## 4. Discussion

This qualitative study explored the perceptions of healthcare providers and volunteers regarding volunteer engagement in a stroke self-management program. Our findings showed that healthcare providers and volunteers have similar views. The findings highlighted a bilateral exchange of information about care after stroke and inspiration from life management wisdom and professional attitudes as key features of the volunteering process; this benefited both volunteers and stroke survivors. Both volunteers and healthcare providers worked in concert to guide and motivate survivors to set and attain goals and personalize strategies to raise survivors’ confidence in self-management. Volunteer engagement can be optimized by developing well-designed programs that clarify volunteers’ roles, strengthen their collaborations with healthcare providers and fulfil their training needs in personalizing care for and communicating with stroke survivors.

Volunteering has been regarded as a mutually beneficial exchange process that can benefit the volunteers in terms of knowledge, skills, enjoyment or a sense of contribution [21]. Consistently, volunteers in our study exchanged information about stroke care with the survivors. In this program, about half of the volunteers were pursuing a health-related discipline and viewed the exchange of information about care after stroke as beneficial to their studies; they also perceived it as offering an opportunity to practise skills such as communication. Similar benefits have been reported in studies examining the perceptions of nursing or medical students participating in volunteer services [22,23,24]. Indeed, volunteering has been increasingly viewed as a supplement to health education, wherein students are allowed to experience real-life problems by working with people with health problems. Such exposure has also been regarded as an alternative teaching and learning tool that can improve student competency, especially given their limited clinical exposure during the coronavirus pandemic [24,25]. Future studies should explore the effects of volunteering on the development of competencies in healthcare students.

We found that the volunteers, while providing encouragement to the survivors, learned from the survivors’ life management wisdom and gained positive momentum in their own lives. Substantial evidence from systematic reviews has documented the multiple physical and psychosocial health benefits associated with volunteering. These benefits are attributable to the emphasis on sharing, generosity and gratitude while serving others, which may improve positive emotions [26,27]. In this study, exchanging life management wisdom may have inspired and motivated the volunteers, thereby increasing positive emotions. This also may be a result of the volunteers spending significant amounts of time talking and sharing with, and hence learning from, the survivors. Indeed, the sharing of survival experiences by survivors has been commonly used as powerful peer support for other stroke survivors as it provides a model experience [10,28]. Narrative therapy has also been shown to help improve stroke survivors’ social and emotional adaptability [29,30]. It focuses on allowing the participants to narrate their own life stories to re-author their lives and reconstruct their identities with new hopes and dreams [29]. It is therefore promising to examine more structured opportunities for survivors to share their life stories, guided by principles of narrative therapy, and enrich mutual gains.

One important finding of this study is the bilateral exchange between the healthcare providers and volunteers not only of health information, caring and communication skills and a sense of empathy, but also intangible benefits such as being inspired by the professional attitudes and passion for serving others in need. This is attributable to over half of the volunteers pursuing a health-related bachelor’s degree and some being practicing healthcare professionals. It would be worthwhile to explore a hybrid team that includes future or even practicing health professionals to increase the benefits of volunteering and support survivors with a group of volunteers with expert knowledge. A more efficient division of labour with volunteer support from the community for stroke survivors would be particularly helpful amidst limited health resources [31].

Goal setting is an important skill in stroke self-management [8,32]. A systematic review of four qualitative studies noted that an individualized and person-centric approach to setting goals is essential to stroke recovery, which is in line with our findings [33]. It is encouraging to note that some volunteers and healthcare providers made concerted efforts to guide and motivate survivors to set and attain goals. It is also important to note that survivors’ needs and expectations are continually being determined and individualized goals and action plans are continuously adjusted, which requires different levels of supervision. A systematic review of nine qualitative studies reported various barriers to effective goal setting: differences in survivors’ and healthcare professionals’ perspectives of goals, survivors’ communication difficulties, cognitive impairments, fatigue and mood disorders and healthcare professionals’ uncertainty regarding the extent of recovery [34]. Volunteer training in goal setting, particularly individually tailored goal-setting, strategies to promote communication and understanding and strategies to avoid disappointment and unrealistic goals should be enhanced [34,35]. Given the diverse educational backgrounds of the volunteers, the training needs to be tailored to ensure that the contents are suited to the volunteers’ duties, but should not overwhelm them.

We found that volunteers can play a significant role in the personalization of strategies to raise survivors’ self-confidence, as stroke recovery is a highly personal and individualized process. It has been reported that the participants of home-based rehabilitation have different primary recovery goals, and that interventions are particularly useful when they relate to the participants’ individual interests [36]. Informed by Bandura’s principles of self-efficacy [37], strategies such as goal setting, sharing videos of recovery after stroke and positive reinforcement were adopted in the program. Some healthcare providers and volunteers faced challenges in fully utilizing and tailoring some strategies, such as providing meaningful positive reinforcement. They required continuous testing with the survivors to find appropriate strategies. The specific tailored strategies identified by previous studies that have adopted this theory for self-management programs have not been summarized [38,39]. Further training in self-efficacy enhancing strategies, driven by theory, and the development of a bank of tailored strategies can help improve volunteer support to the survivors.

Consistent with other qualitative studies on volunteer-supported stroke programs, inconsistent volunteer attendance, inadequate role clarity and poor collaboration between the volunteers and project team were common barriers to the effective engagement of volunteers [14]. To develop effective volunteer-supported self-management programs, it is crucial to integrate attributes that facilitate volunteer engagement. Importantly, volunteer roles should be considered highly valuable and meaningful to improve volunteer support, commitment and attendance. Their roles and responsibilities need to be clarified to ensure continuity of service. This can be achieved by providing the essential knowledge and skills vital for volunteers in the training sessions, enabling them to appropriately perform their delineated responsibilities [14]. Topics such as information about available community support services, caring skills to provide psychological support and effective communication skills for survivors with dysphasia or aphasia are needed. Moreover, in line with our findings, an umbrella review of 39 systematic reviews found that volunteer performance could be strengthened by regular in-service training and supportive supervision [31]. The importance of collaboration between volunteers and healthcare providers should be highlighted to ensure that the survivors’ progress is adequately monitored. Given the diverse health condition of the participating stroke survivors, it is important to provide volunteers with the knowledge and skills in assessing general health conditions, physical mobility, psychological well-being and performance of self-management behaviors using objective measuring scales or questionnaires as adopted in the program [17].

Our findings highlight the benefits of including stroke survivors as volunteers in such programs. This is supported by a systematic review of 12 studies, which showed that peer support promotes social comparison and vicarious learning in survivors, thereby facilitating learning in self-management interventions [28]. Indeed, COMBO-KEY used videos by stroke survivors who shared their survival experiences to provide model experiences. Given the mobility challenges faced by survivors, videos provide flexibility and convenience in seeking model advice from others with similar conditions. Further research should examine how self-management programs can integrate live model experiences from stroke survivor volunteers to better support survivors. 

## 5. Limitations

This study was limited by a relatively small sample size; the sample was restricted to those who volunteered in COMBO-KEY. Moreover, about one third of the volunteers helped support less than three stroke survivors in the program. All of the stroke survivors were cognitively intact, and most of them were able to walk with mild assistance, which may have influenced their perceptions of their volunteering experiences.

## 6. Conclusions

The findings of this study highlight that volunteer-assisted stroke self-management programs entail a bilateral exchange of knowledge and support between healthcare providers/volunteers and survivors. They also encourage the adoption of an individualized, goal-oriented and confidence-enhancing approach. Volunteer engagement can be optimized by developing well-designed programs that sufficiently clarify volunteers’ roles, strengthen their collaborations with healthcare providers and fulfil their training needs in caring for and communicating with stroke survivors. Future research should evaluate the use of peer and healthcare professional volunteers in self-management programs and thereby build community capacity to support the recovery of stroke survivors.

## Figures and Tables

**Table 1:** Demographic characteristics of the participants.

Participant	Age	Gender	Education level	Study Major	Occupation	Role in Program
P1	39	F	Doctoral	Nursing	Registered nurse	Healthcare provider
P2	39	F	Doctoral	Nursing	Registered nurse	Healthcare provider
P3	45	F	Master	Nursing	Registered nurse	Healthcare provider
P4	25	F	Bachelor	Gerontology	Social worker	Healthcare provider
P5	28	M	Bachelor	Social work	Social worker	Healthcare provider
P6	21	M	Bachelor	Nursing	Student	Volunteer
P7	25	F	Bachelor	Nursing	Student	Volunteer
P8	22	M	Bachelor	Nursing	Student	Volunteer
P9	26	F	Bachelor	Psychology	Research assistant	Volunteer
P10	22	F	Bachelor	Nursing	Student	Volunteer
P11	22	F	Bachelor	Nursing	Student	Volunteer
P12	22	F	Bachelor	Nursing	Student	Volunteer
P13	21	F	Bachelor	Gerontology	Student	Volunteer
P14	25	F	Master	Social work	Social worker	Volunteer
P15	22	M	Bachelor	Gerontology	Student	Volunteer
P16	28	F	Bachelor	Nursing	Registered nurse	Volunteer
P17	28	F	Bachelor	Nursing	Registered nurse	Volunteer
P18	28	F	Bachelor	Nursing	Registered nurse	Volunteer
P19	50	F	Bachelor	Nursing	Clerk	Volunteer
P20	25	F	Master	Psychology	Research assistant	Volunteer
P21	26	F	Bachelor	Nursing	Student	Volunteer
P22	22	F	Bachelor	Psychology	Student	Volunteer
P23	50	M	Bachelor	Psychology	Clerk	Volunteer

**Table 2:** Questions in the semi-structured interview guide.

Could you share your experience of volunteering in the program?
2. Which parts of the program do you think are most helpful to stroke survivors?
3. What were the challenges faced when you worked with the volunteers/volunteered in the program?
4. How did you address the challenges faced?
5. How would you describe any gains that you have made from the program?
6. Would you like to continue to work with the volunteers/volunteer in the program and the reasons?
7. How would you comment on your collaboration with the volunteers/healthcare providers when supporting a stroke survivor in the program?
8. What are your suggestions to improve the program and better support the stroke survivors’ self-management?
9. Are there any issues that we have not covered and you would like to share?

**Table 3:** Themes and subthemes that emerged from the interview data.

Themes and Subthemes
Theme 1: Bilateral exchange between survivors and healthcare providers/volunteers
Subtheme 1.1: Exchanging information about care after stroke
Subtheme 1.2: Being inspired by life management wisdom and professional attitudes
Theme 2: Concerted efforts in guiding and motivating survivors
Subtheme 2.1: Keeping survivors on track with their goals
Subtheme 2.2: Personalizing care to raise confidence
Theme 3: Suggestions for optimizing the contributions of volunteers
Subtheme 3.1: Clarifying the importance of roles and collaboration
Subtheme 3.2: Fulfilling training needs in caring for survivors

## Data Availability

The datasets used and/or analyzed during the current study are available from the corresponding author (SHSL) on reasonable request.
